# The role of perfluorooctane sulfonic acid (PFOS) exposure in inflammation of intestinal tissues and intestinal carcinogenesis

**DOI:** 10.3389/ftox.2023.1244457

**Published:** 2023-08-15

**Authors:** Jerika Durham, Josiane Weber Tessmann, Pan Deng, Bernhard Hennig, Yekaterina Y. Zaytseva

**Affiliations:** ^1^ Department of Toxicology and Cancer Biology, University of Kentucky, Lexington, KY, United States; ^2^ College of Pharmaceutical Sciences, Soochow University, Suzhou, China; ^3^ Department of Animal and Food Sciences, University of Kentucky, Lexington, KY, United States

**Keywords:** per- and polyfluoroalkyl substances (PFAS), perfluorooctane sulfonic acid (PFOS), ulcerative colitis, gastrointestinal tract, colorectal cancer

## Abstract

PFAS (per- and polyfluoroalkyl substances) are organofluorine substances that are used commercially in products like non-stick cookware, food packaging, personal care products, fire-fighting foam, etc. These chemicals have several different subtypes made of varying numbers of carbon and fluorine atoms. PFAS substances that have longer carbon chains, such as PFOS (perfluorooctane sulfonic acid), can potentially pose a significant public health risk due to their ability to bioaccumulate and persist for long periods of time in the body and the environment. The National Academies Report suggests there is some evidence of PFOS exposure and gastrointestinal (GI) inflammation contributing to ulcerative colitis. Inflammatory bowel diseases such as ulcerative colitis are precursors to colorectal cancer. However, evidence about the association between PFOS and colorectal cancer is limited and has shown contradictory findings. This review provides an overview of population and preclinical studies on PFOS exposure and GI inflammation, metabolism, immune responses, and carcinogenesis. It also highlights some mitigation approaches to reduce the harmful effects of PFOS on GI tract and discusses the dietary strategies, such as an increase in soluble fiber intake, to reduce PFOS-induced alterations in cellular lipid metabolism. More importantly, this review demonstrates the urgent need to better understand the relationship between PFOS and GI pathology and carcinogenesis, which will enable development of better approaches for interventions in populations exposed to high levels of PFAS, and in particular to PFOS.

## 1 Introduction

PFAS (per- and polyfluoroalkyl substances) are synthetic compounds made of fluorinated carbon chains attached to functional groups (such as alcohols, carboxylic and sulfonic acids, etc.,) (Buck et al., 2011). Because the carbon–fluorine bond is one of the strongest bonds in organic chemistry, natural processes such as hydrolysis, photolysis, microbial degradation, and vertebrate metabolism are ineffective in breaking down PFAS, and these compounds are defined as persistent organic pollutants (Perez et al., 2013; Liang et al., 2022).

PFAS are commercially used for their heat-resistant properties. PFAS are found in firefighting foams, non-stick cookware coatings, food packaging, personal care products, waterproof fabrics, cosmetics, etc., (Xiao, 2017; Zeng et al., 2019). During production and use, PFAS can contaminate soil, water, and air. Over recent years, PFAS have frequently been found in environmental samples, wildlife, and human tissues (Fenton et al., 2021). According to data from the National Health and Nutrition Examination Survey, four PFAS (PFOS or perfluorooctane sulfonic acid, PFOA or perfluorooctanoic acid, PFHxS or perfluorohexane sulfonic acid, and PFNA or perfluorononanoic acid) are found in the serum of nearly every person tested since 1999, indicating widespread exposure in the U.S. (Centers for Disease Control and Prevention, 2022). Environmental concentrations and human exposures to PFAS are typically highest at contaminated sites, linking PFAS to potential adverse health outcomes (De Silva et al., 2021).

PFAS are often divided into two groups, long and short chain (Buck et al., 2011). Long-chain PFAS have comparable bioaccumulation potential as other well-known contaminants such as polychlorinated biphenyls (PCBs) and dichlorodiphenyltrichloroethane (DDT) (American Water Works Association, 2019; Shahsavari et al., 2020). PFOS is a “long-chain” subtype of PFAS. In 2009, PFOS was added to the United Nations Stockholm Convention’s list of Persistent Organic Pollutants (Stockholm Convention, 2019). According to The National Health and Nutrition Examination Survey biomonitoring data, the median and 95% percentile of blood serum PFOS levels in general population are 2 ng/mL and 4 ng/mL, respectively (U.S. Environmental Protection Agency, 2019). The elimination half-life of PFAS in humans is roughly estimated to be between 1.5 and 4.8 years, with PFOS elimination approximately 4.8 years (Olsen et al., 2007; Post et al., 2012). The long half-life of PFOS in humans means that cumulative exposures over a relatively long time period can significantly influence human health (Buck et al., 2011). PFOS has been associated with multiple negative health outcomes such as hepatotoxicity, neurotoxicity, reproductive toxicity, immunotoxicity, thyroid disruption, cardiovascular toxicity, pulmonary toxicity, renal toxicity, and carcinogenesis (Zeng et al., 2019; Chen et al., 2020; Fenton et al., 2021).

The contribution of PFAS to cancer has been studied in multiple animal models, and several studies reported that administration of PFAS such as PFOA and PFOS is associated with development of testicular Leydig cell adenomas, pancreatic acinar cell adenomas, and hepatocellular adenomas or carcinomas in rats (Fenton et al., 2021). However, there are some concerns regarding the relevance of these studies to humans, and epidemiological studies are thought to be more valuable in providing insight into potential links between PFAS exposure and cancer.

The gastrointestinal (GI) tract is one of the systems that is exposed to high concentrations of environmental pollutants via contaminated drinking water and food. PFOS is one the most frequently detected PFAS in drinking waters and its levels have been above the method reporting limit (40 ng/L) in drinking water in approximately 1.9% of U.S. Public Water Systems (American Water Works Association, 2019). PFOS is readily absorbed in the gastrointestinal tract and distributes predominantly to the plasma and liver. It is not metabolized and is excreted in both urine and feces (EFSA Panel on Contaminants in the Food Chain et al., 2018).

Scientific studies on PFOS and GI are limited and the association between this environmental pollutant and GI-associated diseases remains poorly understood. The scientific literature includes contrasting studies related to the association of PFOS, inflammatory bowel disease (IBD), and GI malignancies. This review provides an overview of current research regarding the contribution of PFOS exposure to processes associated with GI inflammatory diseases and colorectal carcinogenesis. It also briefly discusses the potential intervention strategies to eliminate the harmful effects of PFOS on the GI tract.

## 2 PFAS/PFOS exposures and population cancer studies

Due to widespread exposure and high bioaccumulation in human tissues, understanding the carcinogenic properties of PFAS has been a high priority for scientific and medical communities for the last decade. It is important to note the complexity of studying and understanding the effects of PFAS in human health. Different PFAS have distinct physical, chemical, and toxicological properties, and since people are most likely to be exposed to mixtures of PFAS, it is difficult to associate the specific health issues with different PFAS species. To address the gaps in our understanding of the carcinogenicity of PFAS, the NIH Division of Cancer Epidemiology and Genetics (DCEG) has launched a series of studies aimed at identifying specific cancers associated with PFAS at exposure levels typically found in the general population (National Cancer Institute, 2017). The risks of developing different cancers have been evaluated based on direct assessment of exposure to multiple PFAS in banked serum specimens. These studies provide sufficient evidence that increasing PFAS exposure has been associated with the development of kidney cancer. Moreover, there is limited or suggestive evidence of PFAS association with breast and testicular cancers. The studies on these and other cancer types are ongoing and aim to inform us of the potential carcinogenic effects of multiple PFAS. Studies have suggested possible PFAS links to other cancers, including thyroid, prostate, bladder, breast, and ovarian cancer, but more research is needed to clarify these findings (Steenland and Winquist, 2021; American Cancer Society, 2023b).

According to the National Academies of Sciences, Engineering, and Medicine (NASEM) report, “Guidance on PFAS Exposure, Testing, and Clinical Follow-Up,” (2022) there is suggestive evidence of carcinogenic potential for PFOS (NASEM, 2022). However, human epidemiology studies found no direct correlation between PFOS exposure and the incidence of carcinogenicity in worker-based populations. The evidence for PFOS exposure and an increase in bladder cancer and associated mortality was supported by a retrospective cohort study of 2083 employees of a perfluorooctanesulphonyl fluoride based fluorochemical production facility (Alexander et al., 2003; Alexander and Olsen, 2007). Consistent with the suggested carcinogenic role of PFOS in the NASEM report, a more recent nested case–control study shows that high PFOS levels (90th percentile from NHANES; >55 μg/L) are associated with a 4.5 fold increased risk of hepatocellular carcinoma (HCC), likely via alterations in glucose, amino acid, and bile acid metabolism (Goodrich et al., 2022). Consistently, another recent study, which analyzed 37 serum and tumor samples from patients with hepatobiliary and gastrointestinal malignancy for 24 analytes of PFAS. Patients with PFOS in tumor samples had significantly higher levels in serum when compared to tumor samples without PFOS (9.4 ng/mL vs. 5.5 ng/mL; *p* = 0.015). The serum PFOS levels were significantly higher in tumor samples when compared to the reported national levels (6.77 ng/mL vs. 5.20 ng/mL; *p* = 0.038) (Kelly-Schuette et al., 2022). In contrast to this study, an inverse association of colorectal cancer (CRC) prevalence to serum levels of PFOS and PFOA in a large Appalachian population exposed to pollutants through contaminated drinking water was reported by Innes et al. (2014). Interestingly, PFAS including PFOS, have also been implicated in development of pediatric cancers. A hospital-based case–control study reported significantly higher serum PFOS and four other PFAS concentrations in pediatric patients with germ cell tumors compared to age- and sex-matched tumor-free pediatric patients (PFOS 3.888 vs. 5.202 ng/mL, *p* = 0.036) (Lin et al., 2020).

In summary, the number of studies on PFAS association with cancer is very limited and more well-designed and controlled general population and occupational cohort studies are needed to understand the contribution of PFAS exposures to different cancer types.

## 3 PFAS/PFOS, IBD, and colorectal cancer

Colorectal cancer (CRC) is the second most common cause of cancer death in the United States (American Cancer Society, 2023a). CRC incidence rates have been declining among those who are 50 years of age or older due to screening efforts. However, the incidence of CRC in young adults under 50 has drastically increased since the 1990 s (Stoffel and Murphy, 2020). The increase in incidence of CRC in younger individuals poses a major public health concern since CRC is currently the leading cause of death in men younger than 50 years (American Cancer Society, 2023a). The causes of rising cases of CRC in younger adults remain elusive but diet, environmental exposures, and lifestyle are thought to play a major contributing role. Environmental pollutants, such as PFAS exposure, could be one of the contributing factors to rising incidence. According to the NASEM report, there is suggestive evidence of an association between PFAS and ulcerative colitis in adults (NASEM, 2022). The published studies show an association between the high PFAS levels in blood and ulcerative colitis (Steenland et al., 2018; Fart et al., 2021). Ulcerative colitis is a chronic IBD in which abnormal reactions of the immune system cause inflammation and ulcers on the inner lining of the large intestine. The risk of CRC is increased among patients with ulcerative colitis (Ekbom et al., 1990; Lakatos and Lakatos, 2008; Kunovszki et al., 2020; Olen et al., 2020). A meta-analysis of population-based cohort studies shows that ulcerative colitis increases the risk of CRC 2.4 fold. Male sex, young age at diagnosis with ulcerative colitis, and extensive colitis increase the risk (Jess et al., 2012). Moreover, a recent study shows that IBD-associated CRC occurs in younger patients and has worse outcomes than sporadic CRCs (Birch et al., 2022). Therefore, PFAS exposure could be one of the causative factors leading to the development of IBD and CRC. However, there is insufficient evidence linking PFAS to the development of CRC. More studies are needed to define the link between PFAS, IBD and cancer to elucidate the contribution of different PFAS to GI pathology. The NASEM report also stated the possible PFOS effects on disease and impairment of the digestive system in general, including gallbladder dysfunction, irritable bowel syndrome, and other colon impairment (NASEM, 2022).

## 4 PFOS exposure and GI carcinogenesis in animal studies

To investigate toxicity and neoplastic potential from chronic exposure to PFOS, a two-year dietary toxicity and cancer bioassay study was conducted with potassium PFOS in male and female Sprague Dawley rats (Butenhoff et al., 2012). Rats were fed with PFOS (concentration range 0.5–20 μg/g) in their diet for up to 104 weeks. Additional groups were fed 20 μg/g for the first 52 weeks, after which they were fed control diet through study termination (recovery groups). These studies demonstrated that extended exposure to PFOS leads to an increase in liver hepatocellular adenomas, pancreatic islet cell carcinomas, and thyroid follicular cell adenomas in male rats, and liver hepatocellular adenoma and carcinoma combined and thyroid follicular cell adenomas and carcinoma in female rats. Statistically significant increases in benign hepatocellular adenoma were observed at the highest dose tested for both males and females. One hepatocellular carcinoma was observed in a female rat with high-dose exposure (Butenhoff et al., 2012). The results of these two-year studies provided strong evidence of potential cancer risk from chronic exposure to PFOS. Interestingly, this study also highlights the gender differences in responses to PFOS exposure and shows differences in survival, hepatocellular adenoma and thyroid follicular cell adenoma incidence in males and females of the same group and time point (Butenhoff et al., 2012). The potential explanations for the observed gender differences seen in Sprague rats following the PFOS exposure are higher PFOS concentrations in serum and liver of females as compared to males and different clinical chemistry such as the level of cholesterol and urea between females and males. Consistent with Butenhoff et al.’s study showing the contribution of PFOS to development of liver tumors in rats, another study demonstrated that six-month dietary exposure to PFOS results in increased liver cancer incidence in rainbow trout (Benninghoff et al., 2012). Scientific literature related to PFOS exposure and CRC is very limited. Two studies using APC57BL/6J-Apc ^Min^ mice prone to familial adenomatous polyposis found that PFOS exposure either had no effect or was protective when it occurred during tumor development (Ngo et al., 2014; Wimsatt et al., 2016). The first study tested PFOS exposure *in utero* and showed that the dams exposed to PFOS (0.01, 0.1 or 3.0 mg/kg bw/day) by oral gavage on GD1-17 did not result in an increase in the incidence or number of tumors in the small intestine or colon of the APC^min^ mice or affect their location along the intestines (Ngo et al., 2014). In another study, APC^min^ mice (5-6 weeks old) received 0, 20, or 250 mg PFOS/kg (females) or 0, 10, 50, or 200 mg PFOS/kg (males) via their drinking water and adenomas were counted/scored and blood PFOS levels measured at 15 weeks of age. The authors of this study suggested that chronic exposure to PFOS in drinking water can reduce formation of GI tumors (Wimsatt et al., 2016). To our knowledge there are no follow-up studies performed in CRC transgenic mouse models to support these results.

In summary, studies on PFOS and carcinogenesis are very limited and demonstrate contradictory results in different cancer types. To recapitulate the chronic exposure to PFAS in humans, the long-time exposure with low doses of PFOS relevant to environmental exposures should be considered in future animal studies.

### 4.1 Potential mechanisms of PFOS-mediated carcinogenesis

**FIGURE 1:**
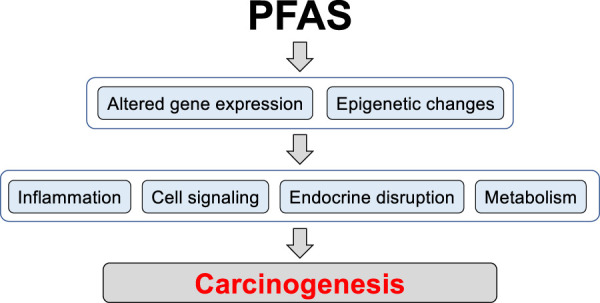
The potential mechanisms of PFAS contribution to carcinogenesis. Long-term PFAS exposures can lead to changes in gene expression and induction of epigenetic alterations including DNA methylation and histone modification, thereby regulating patterns of gene expression. Changes in chromatin structure and gene expression can enhance activity of signaling pathways involved in pro-inflammatory and pro-carcinogenic processes, induce endocrine disruption, and rewire metabolism to support pro-carcinogenic program in cells.

Current literature suggests that PFAS, including PFOS, can contribute to carcinogenesis via alteration of gene expression and epigenetic changes, thus affecting multiple signaling pathways in different cell types (Figure 1). The published studies on PFOS exposures in *in vitro* and *in vivo* cancer models are summarized in Table 1.

**TABLE 1:** Published *in vitro* and *in vivo* studies on PFOS exposure and cancer.

Reference	Model		PFOS exposure time		PFOS exposure concentration		Main findings
	*In vitro*	*In vivo*	*In vitro*	*In vivo*	*In vitro*	*In vivo*	
Breast							
Sonthithai et al. (2016)	T47D	--	24 or 48 h	--	0.001, 0.01, and 0.1 μM	--	↑ 17β-estradiol effect on estrogen-responsive gene expression and proliferation
Pierozan and Karlsson (2018)	MCF-10A	--	72 h	--	10 μM	--	↑ proliferation, cell cycle alteration, ↑ migration, ↑ invasion; no effect on Erα and Erβ levels
Pierozan et al. (2020)	MCF-10A	--	72 h	--	10 μM	--	↑ proliferation, cell cycle alteration, ↑ migration, ↑ invasion and epigenetic modifications
Pierozan et al. (2023)	MCF-10A	--	72 h	--	10 μM (binary mixture of PFOS and PFOA)	--	↑ proliferation, alteration of regulatory cell-cycle proteins, ↑ migration, ↑ invasion and epigenetic modifications
Colorectal							
Ngo et al. (2014)	--	APC^min^ mice	--	GD 1 to GD 17, the analyses were performed on the offspring at week 11	--	0.01, 0.1 or 3 mg/kg bw/day–by oral gavage	No association with number of small intestinal or colonic tumors
Wimsatt et al. (2016)	--	APC^min^ mice	--	8 (females) or 9 weeks (males)	--	20, 250 (females) or 10, 50, 200 mg/kg (males)–in drinking water	↓ tumor number
Wimsatt et al. (2018)	--	PDX	--	10 weeks	--	100 mg/kg–in drinking water	↓ tumor size
Esophagus							
Liu et al. (2022)	HEEC, KYSE150, KYSE140 and KYSE70	--	24 h	--	10 nM	--	↑ migration and ↑ invasion in carcinoma cells via upregulation of transcription and protein stability of ZEB1
Glioblastoma							
Merritt and Foran (2007)	T98G		5 days		0.005, 0.01, 0.5, and 1 μM		↑ proliferation
Kidney							
Liu et al. (2023)	A498	--	24 or 48 h	--	50 μM	--	↑ viability and epigenetic modifications
Liver							
Benninghoff et al. (2012)	--	Rainbow trout	--	6 months	--	2.5 mg/kg/day	↑ tumor incidence
Zhu et al. (2021)	--	Kras^V12^ transgenic zebrafish	--	10 days	--	500 μg/L PFOS	↑ tumor incidence and changes in metabolic pathways in Kras^V12^-induced zebrafish
Lung							
Jabeen et al. (2020)	A549		24 or 48 h		10, 200, and 400 μM		↑ proliferation in low doses, epigenetic modifications, cell cycle alteration and dose-dependent induction of apoptosis
Prostate							
Imir et al. (2021)	RWPE-1 and RWPE-KRAS	Xenograft model with RWPE-KRAS	1 week or 24 h	5 days/week for 40 days	10 nM	10 mg/kg–by oral gavage	↑ tumor growth, synergistic effect with high fat diet to increase tumor burden, changes in metabolic phenotype, and epigenetic modifications
Hu et al. (2022)	Stem-progenitor cells from donors and RWPE-KRAS	Xenograft model with RWPE-KRAS	3-4 weeks	40 days	10 nM	10 mg/kg bw/day–by oral gavage	↑ stem cell self-renewal, alters luminal progenitor cell differentiation, changes stem-progenitor cell transcriptome and metabolome and ↑ tumor growth

#### 4.1.1 PFOS and inflammation

Chronic inflammation increases the risk for different types of cancers, including CRC, liver, bladder, pancreatic, and esophageal cancers (Multhoff et al., 2011). In particular, patients with IBD, categorized into ulcerative colitis and Crohn’s disease, are two to six times more likely to develop CRC compared to the general population (Keller et al., 2019). In addition, aspirin, a non-steroidal anti-inflammatory drug, is considered a potential intervention for the prevention of CRC, supporting the concept that chronic inflammation is linked to tumorigenesis (Drew et al., 2016).

Evidence in the literature suggests that PFOS can promote chronic inflammation by inducing an increase in pro-inflammatory cytokine production. Although some data are still controversial, the most well-represented cytokines are TNF-α, IL-6, and IL-1β (Temkin et al., 2020; Elmore et al., 2021; Zhang et al., 2023). A study by Diaz et al. determined that PFOS enhanced pro-inflammatory cytokine expression in zebrafish and mice. Specifically, they showed that exposure to PFOS during 2,4,6-trinitro-benzene sulfonic acid (TNBS)-induced inflammation enhanced the expression of proinflammatory cytokines such as IL-1β and TNF-α as well as neutrophil recruitment to the intestine of zebrafish larvae. These findings were validated in the TNBS-induced colitis mouse model. Moreover, PFOS exposure in mice undergoing colitis resulted in neutrophil-dependent increased intestinal permeability and enhanced PFOS translocation into the circulation. This was associated with a neutrophil-dependent expansion of systemic CD4^+^ T cells. Thus, their results indicate that PFOS worsens inflammation-induced intestinal damage with disruption of T-cell homeostasis (Diaz et al., 2021). Alteration of intestinal barrier integrity and an increase in pro-inflammatory cytokine production are the characteristics of IBD, further suggesting that PFOS exposure can contribute to development and severity of this disease. Consistent with the Diaz et al. study, a publication by Liang et al. showed that high doses of PFOS (10 mg/kg) induced an inflammatory bowel phenotype in rats (Liang et al., 2021). They demonstrated that PFOS exposure over time increased rat body weight, and induced markers of intestinal inflammation, higher histopathological score in intestinal tissues, and apoptosis in the proximal jejunum. Interestingly, neutrophil and macrophage accumulation and inflammatory cytokine infiltration were also remarkably increased in rats exposed to PFOS. The results of the study suggest that PFOS can induce an inflammatory bowel-like phenotype in rats.

It is well established that an inflammatory microenvironment can contribute to different stages of cancer development and progression (Schmitt and Greten, 2021). During chronic inflammation, pro-inflammatory cells such as macrophages and neutrophils are potent sources of reactive oxygen species (ROS) and reactive nitrogen species (RNS). These molecules can induce cellular damage, such as mutations in tumor suppressor genes and modifications in proteins involved in DNA repair, apoptosis, and cell cycle checkpoint, contributing to tumor initiation (Hussain and Harris, 2007). In addition, inflammatory signaling, mainly through cytokines such as IL-6 and TNF-α, leads to epigenetic changes (Grivennikov, 2013). Among them, DNA methylation, histone modification, microRNAs, and lncRNAs modulate the expression level of oncogenes and tumor suppressor genes that contribute to the occurrence and development of inflammation-induced CRC (Yang et al., 2019). Another mechanism linked to tumor initiation is related to stem cells. An inflammatory environment can induce de-differentiation of non-stem cells into tumor-initiating stem-like cells. Furthermore, particularly in intestinal cells, inflammation affects epithelial barrier function, which can expose the stem cell compartment to environmental carcinogens or molecules released by active inflammatory cells (Greten and Grivennikov, 2019).

The inflammatory cells act as a source of cytokines and growth factors to promote tumor cell survival and proliferation (Hibino et al., 2021). The main pathways involved in this process are NF-κB, MAPK, JAK-STAT, and PI3K-AKT pathways (Zhao et al., 2021). In addition, the action of cytokines, such as TNF-α, IL-6, and IL-1β, in tumor progression includes the recruitment of immuno-suppressive cells and induction of angiogenesis and metastases (Kartikasari et al., 2021).

Upregulation of inflammatory responses due to PFOS exposure is also supported by studies reporting inflammasome activation and subsequent cytokine release by pyroptotic cells (Zhang et al., 2023). For instance, the mechanism associated with PFOS-induced lung inflammation in offspring rats was upregulation of inflammasome-associated proteins, such as NLRP3, ASC, Caspase-1, and GSDMD, and an increase in inflammatory cytokines IL-18 and IL-1β (Zhang et al., 2021). Corroborating this study, Qin et al. (2022) found similar mechanisms resulting in liver inflammation and steatosis after exposure to PFOS (Qin et al., 2022). Alternatively, PFOS-mediated inflammatory response and tissue damage may also be independent of the NLRP3 inflammasome. Instead, PFOS activated AIM2 inflammasome, a sensor to recognize exogenous or endogenous double-stranded DNA, through mitochondrial dysfunction and release of mitochondrial DNA (mtDNA). Activation of AIM2 led to IL-1β production and pyroptosis in macrophages (Wang et al., 2021). These reports highlight the involvement of the innate immune system and the possible mechanism of how PFOS can be recognized in cells and trigger cellular inflammatory responses.

Inflammasome activation may influence CRC induction and progression due to its central role in promoting inflammation. On the other hand, it may have an anticancer effect by triggering pyroptosis and immunoregulatory functions (Keshavarz Shahbaz et al., 2021).

#### 4.1.2 PFOS and immune environment

Another mechanism by which PFOS exposure may be related to tumorigenesis is through its ability to decrease immune system function, leading to immunosuppression. Some studies have reported a reduction in circulating immunoglobulins, such as IgM and IgG, as well as a decrease in the activity or proliferation of natural killer (NK) cells, B cells, and CD4^+^ CD8^+^ T-cell subtypes (Temkin et al., 2020; Elmore et al., 2021; Zhang et al., 2023). The immune system plays a crucial role in preventing tumor development. It can protect the host against virus-induced tumors by suppressing viral infections. In addition, the immune system controls inflammation and thus prevents an environment conducive to tumorigenesis. Ultimately, the immune system identifies and controls nascent tumor cells through immunosurveillance (Swann and Smyth, 2007).

Immunosurveillance is the initial step of an emerging concept known as immunoediting. This process involves three sequential phases. In the first, the elimination phase, immune cells, such as CD8^+^, CD4^+^, NK cells, and macrophages, identify tumor-associated antigens resulting in the elimination of tumor cells. If this process is not completed, the second phase is initiated, which corresponds to a state of equilibrium where tumor cells and immune cells coexist. Finally, the escape phase, wherein the immune system fails to eliminate the tumor resulting in the selection of tumor cell variants with a greater ability to resist or suppress the immune system (Dunn et al., 2002; Swann and Smyth, 2007; Kunimasa and Goto, 2020).

NK cells have a central function in tumor immunosurveillance. Tumor cells can be recognized by NK cells through stress-induced ligands or a decrease in the levels of major histocompatibility complex (MHC) I. Upon activation, NK cells have a cytotoxic function and can modulate innate and adaptative immunity through the secretion of cytokines and chemokines (Morvan and Lanier, 2016; Wang et al., 2021). A study showed that low NK cell activity is correlated with a higher risk of developing various types of cancer (Imai et al., 2000). In CRC, low NK cell infiltration or activity is associated with poor overall survival and relapse after treatment (Fionda et al., 2022). Indeed, high frequency of metastasis-infiltrating NK and T cells is associated with better overall survival (Donadon et al., 2017). These cells may also play a role in CRC development since their presence in tissues from early stages is scarce (Halama et al., 2011).

#### 4.1.3 PFOS and metabolic alterations

Several studies have reported that PFOS exposure can induce oxidative stress (Temkin et al., 2020; Elmore et al., 2021). This phenomenon is defined by an imbalance between ROS generation and their elimination by the biological antioxidant system, resulting in oxidative damage to cellular components such as DNA, lipids, and proteins (Pizzino et al., 2017). In addition to its role in carcinogenesis by directly damaging these components, ROS can contribute to tumor survival and progression through activation of signaling pathways such as ERK1/2, p38/MAPK, BMK1, and PI3K/AKT. Furthermore, through the activation of protein kinases, inactivation of phosphatases or by direct redox reaction, ROS regulate transcription factors such as Nrf2, NF-κB, HIF, p53, AP-1, FOXO, STATs, and Smad. These events may contribute to cell survival, increased proliferation, invasion, metastasis, angiogenesis, apoptosis suppression, and cancer stem cell survival (Ding et al., 2015; Zhang et al., 2016; Gao and Schottker, 2017; Kumari et al., 2018; Aggarwal et al., 2019). PFOS has been associated with induction of oxidative DNA damage, generation of ROS or RNS, lipid peroxidation, increased levels of Nrf2, and alteration of levels or activity of antioxidant enzymes (Temkin et al., 2020; Elmore et al., 2021).

PFOS has been found to cause metabolic perturbation. Although the current data are still inconsistent, PFOS was positively associated with an increase in total serum cholesterol, and in some cases, triglycerides in human epidemiological studies (Fragki et al., 2021). Another study reported that PFOS exposure increased expression levels of enzymes involved in peroxisomal fatty acid β-oxidation (Tan et al., 2012). Accordingly, Hu et al. (2005) showed that PFOS disturbs the expression of genes associated with fatty acid (FA) metabolizing enzymes, cytochrome P450s, and genes involved in hormone regulation. PFOS also induced deregulation in amino acid and glucose metabolism (Kariuki et al., 2017; Lai et al., 2018; Alderete et al., 2019).

Due to its fluorine-saturated carbon backbone and a charged moiety at one end, PFOS resemble the structure of FAs (Wolf et al., 2008). A suggested biological effect induced by PFOS is the activation of peroxisome proliferator activated receptors (PPARs). PPARs are a type of nuclear receptor that upon ligand-binding, such as FAs, heterodimerize with retinoid X receptors (RXRs) and act as transcription factors. There are three isoforms of PPARs, namely, PPARα, PPARβ/δ, and PPARγ (Grygiel-Gorniak, 2014). All isoforms control a group of genes involved in lipid and glucose metabolism, adipogenesis, and inflammation (Fragki et al., 2021). Several studies have shown that PFOS can primarily activate PPARα. However, its activation seems to be weaker in humans compared to mice. Although less reported, it is also possible that PFOS may have a role in the regulation of PPARγ (Wolf et al., 2008; Elmore et al., 2021; Boyd et al., 2022). PPARα and PPARγ are generally associated with anti-cancer effects, such as decreased inflammation, cell survival, glycolysis, and metastatic potential, as well as induction of cell differentiation and apoptosis. However, the activation of these receptors also has pro-cancer functions, including maintaining cancer stemness, improving glucose and lipid metabolism, which supplies the high energy demands of cancers, and promoting metastasis (Cheng et al., 2021; Chi et al., 2021). A study revealed that PFOS increased the self-renewal capacity of prostate epithelial stem-progenitor cells (SPCs), induced upregulation of cancer-associated signaling pathways, and enhanced glycine and serine metabolism, as well as glucose metabolism through the Warburg effect. Upon stimulation by PFOS, SPC increased expression of PPARs and RXRs that likely mediate its effects in promoting a pre-malignant stem-progenitor cell fate (Hu et al., 2022). Another report showed that PFOS caused a decrease in hepatocyte nuclear factor 4-alpha (HNF4α) protein expression in human hepatocytes and promoted changes in the expression of genes involved in lipid metabolism and carcinogenesis. The authors highlighted the role of HNK4α in maintaining differentiation; therefore, its loss may contribute to hepatocellular de-differentiation and carcinoma development (Beggs et al., 2016).

#### 4.1.4 PFOS and epigenetic changes

Other important pathways linked to tumor initiation and progression are epigenetic mechanisms, which refer to changes in gene expression without altering DNA sequence (Lu et al., 2020). These modifications can inappropriately activate oncogenes and/or inhibit tumor suppressor gene expression through DNA methylation, histone modification, and non-coding RNAs (Jones and Baylin, 2002; Lu et al., 2020). All these types of modifications have been associated with PFOS exposure (Temkin et al., 2020; Elmore et al., 2021). Epidemiological studies identified a significant association between PFOS and epigenetic alterations in both adult and birth cohorts (Kim et al., 2021). However, the molecular link between epigenetic changes and PFOS in relation to cancer has been less studied. Additionally, PFOS has been associated with an increase in xenograft prostate tumors, as well as an increase in glucose and pyruvate metabolism. This effect was enhanced when mice were fed a high-fat diet. Further analyses suggested that PFOS has a synergistic effect with a high-fat diet to activate PPARα. This alters the cellular metabolome and increases histone acetylation, which may drive tumor growth and progression (Imir et al., 2021). Another study, using super-resolution imaging and machine-learning tools, revealed that PFOS can alter the spatial organization of the repressive heterochromatin marks H3-lysine-9-trimethylation (H3K9me3) and H3-lysine-27-trymethylation (H3K27me3) in kidney cancer cells. PFOS also upregulated the expression levels of histone demethylase KDM4A. These alterations may be a potential driver of PFOS-induced toxicity in kidney disease development (Liu et al., 2023). Pierozan et al. (2020) showed that PFOS increased the proliferation of MCF-10A, altered the expression of proteins related to cell cycle, and promoted cell migration and invasion. These changes may involve epigenetic mechanisms, as PFOS induced an increase in global methylation and altered important histone modifications. Wang et al. (2015) reported that PFOS can induce changes in miRNA expression, which can target oncogenes and tumor suppressor genes. Thus, PFOS potentially alters pathways associated with different types of tumors, such as melanoma, pancreatic, CRC, and glioma. Corroborating this, analysis of livers from rats exposed to PFOS showed that most up- and downregulated miRNAs were involved in the regulation of genes associated with the epithelial–mesenchymal transition (EMT) process. This is an important phenomenon involved in tumor development, progression, and metastasis (Dong et al., 2016).

### 4.2 Potential intervention strategies for dietary PFOS exposure

PFOS exposure is a major public health concern that affects people all over the world (Fenton et al., 2021). Humans are exposed primarily through consumption of contaminated water and food (Roth et al., 2020). Chemical engineering controls and clean-up efforts are still in development; however, the financial burden of these controls is high. Other strategies to mitigate negative health effects of PFOS are needed to improve overall quality of life for individuals who live in PFOS-contaminated areas. Less expensive mitigation strategies, such as dietary intervention strategies, could potentially be beneficial (Roth et al., 2020). The article by Deng et al. (2022) showed that soluble diets, such as inulin and pectin, have protective effects against negative consequences of PFOS in mice. They suggested that the beneficial effects of soluble fiber diets could be attributed to their ability to be metabolized by and modulate the microbiome population (Deng et al., 2022). Further, they believe that the microbiome population could affect host metabolism by producing bioactive metabolites such as short-chain fatty acids (SCFAs). Mice fed inulin and pectin produce fatty acid species which include acetate, propionate, and butyrate. However, inulin is the preferred source to produce more propionate and butyrate than pectin. The mice receiving inulin also had higher propionate in the portal vein, which could potentially reduce lipogenic enzymes and fatty acid synthesis (Deng et al., 2022). The production of bioactive metabolites such as SCFAs can improve gut barrier integrity, glucose, and lipid metabolism. Bioactive metabolites can also assist with regulation of the immune system, inflammatory response, and blood pressure. Bioactive metabolites could also affect the gut–brain axis and improve immune activation, intestinal permeability, enteric reflex, and entero-endocrine signaling (Mayer et al., 2022). Lipid species were also altered following PFOS exposure; however, mice that received the soluble fiber diets had some protective effects against PFOS-induced alterations in lipid species. In the study, ceramide species (such as Cer 24:1, Cer 16:0, and Cer 22:0), were upregulated in PFOS-exposed mice. Ceramide and sphingomyelin ratios were lower in inulin fed mice compared with mice receiving the control diet. Sphingolipid hydrolysis by sphingomyelinase can produce ceramide through a biosynthesis pathway. The ceramide/sphingolipid ratio can represent the rate of this biosynthesis pathway and enzymatic activity of sphingomyelinase. Enzymatic activity was higher in the PFOS-exposed group; however, inulin feeding partially reduced this effect (Deng et al., 2022). The data from this study suggest that inulin can possibly reduce ceramide accumulation. Fiber diets used in the study by Deng and others represented about a 60% increase in fiber contents compared to standard chow and are representative of recommendations provided for human fiber intake. For comparison, the recommendation for human dietary intake is at least a 50% increase in fiber contents according to the European Food Safety Authority and the U.S. Institute of Medicine. Mice that received the inulin diet had higher Bifidobacterium, which are important for the promotion of dietary fiber digestion, production of vitamins and other chemicals, and infection prevention. Bifidobacterium is a type of probiotic that is good for gastrointestinal tract health. Mice receiving the pectin diet had higher Duncaniella and the effects of this species on gut health are still under investigation. Data from the study suggest that PFOS can alter microbial structure by interacting with a dietary component (Deng et al., 2022). The study by Dzierlenga and others also proposed a mechanistic theory for fiber induced PFOS elimination. A study by Dzierlenga et al. (2021) suggests that dietary fiber increases the gastrointestinal excretion of PFOA, PFOS, and PFNA. The increase in excretion of PFAS substances can lead to a reduction in serum PFAS levels. Increased clearance of PFAS substances could be beneficial to reduce carcinogenicity and other possible long-term effects. The extended half-life of PFAS substances in the human body poses a critical public health concern. Fiber-induced clearance of these “forever chemicals” could vastly improve negative long-term PFAS outcomes by decreasing the amount of time PFAS are in the body. Therefore, the gut-microbiome interaction with other dietary components could also play a key role in the dietary elimination and immune clearance of PFOS from the gastrointestinal tract and intestinal recovery.

## 5 Conclusion

The evidence for an association between PFOS and GI cancer remains sparse. Weaknesses in human and animal study design and methods can lead, in some cases, to contradictory results. Current literature suggests a link between long-term PFOS exposure, lipid metabolism dysregulation, inflammation, microbiome dysfunction and the etiology of colorectal cancer. Most importantly current studies propose that healthful dietary interventions (e.g., high fiber diets) can reduce or prevent PFOS-mediated disease risks. Thus, further mechanistic studies are critically needed to better understand the contribution of PFOS exposure to GI pathology. Careful design of animal studies using long-term exposure and physiologically relevant doses of PFOS should be considered to delineate the mechanisms how PFOS may contribute to GI pathology and carcinogenesis. Analysis of data from large cohorts with a wide range of exposures and long-term follow-up is most likely to provide further insight into the contribution of PFOS exposure to human health, including the GI system.

## References

[B1] Aggarwal V. Tuli H. S. Varol A. Thakral F. Yerer M. B. Sak K. (2019). Role of reactive oxygen species in cancer progression: molecular mechanisms and recent advancements. Biomolecules 9 (11), 735. 10.3390/biom9110735 31766246 PMC6920770

[B2] Alderete T. L. Jin R. Walker D. I. Valvi D. Chen Z. Jones D. P. (2019). Perfluoroalkyl substances, metabolomic profiling, and alterations in glucose homeostasis among overweight and obese hispanic children: a proof-of-concept analysis. Environ. Int. 126, 445–453. 10.1016/j.envint.2019.02.047 30844580 PMC6555482

[B3] Alexander B. H. Olsen G. W. (2007). Bladder cancer in perfluorooctanesulfonyl fluoride manufacturing workers. Ann. Epidemiol. 17 (6), 471–478. 10.1016/j.annepidem.2007.01.036 17448680

[B4] Alexander B. H. Olsen G. W. Burris J. M. Mandel J. H. Mandel J. S. (2003). Mortality of employees of a perfluorooctanesulphonyl fluoride manufacturing facility. Occup. Environ. Med. 60 (10), 722–729. 10.1136/oem.60.10.722 14504359 PMC1740403

[B5] American Cancer Society (2023a). Cancer statistics, 2023. Available At: https://acsjournals.onlinelibrary.wiley.com/doi/full/10.3322/caac.21763 (Accessed May 31, 2023).

[B6] American Cancer Society (2023b). Perfluorooctanoic acid (PFOA), perfluorooctane sulfonate (PFOS), and related chemicals Available At: https://www.cancer.org/healthy/cancer-causes/chemicals/teflon-and-perfluorooctanoic-acid-pfoa.html [Accessed 31 May 2023].

[B7] American Water Works Association (2019). Per- and polyfluoroalkyl substances: overview and prevalence. Available At: https://www.awwa.org/Portals/0/AWWA/ETS/Resources/Per-andPolyfluoroalkylSubstances(PFAS)-OverviewandPrevalence.pdf?ver=2019-08-14-090234-873 (Accessed May 26, 2023).

[B8] Beggs K. M. McGreal S. R. McCarthy A. Gunewardena S. Lampe J. N. Lau C. (2016). The role of hepatocyte nuclear factor 4-alpha in perfluorooctanoic acid- and perfluorooctanesulfonic acid-induced hepatocellular dysfunction. Toxicol. Appl. Pharmacol. 304, 18–29. 10.1016/j.taap.2016.05.001 27153767 PMC5367386

[B9] Benninghoff A. D. Orner G. A. Buchner C. H. Hendricks J. D. Duffy A. M. Williams D. E. (2012). Promotion of hepatocarcinogenesis by perfluoroalkyl acids in rainbow trout. Toxicol. Sci. 125 (1), 69–78. 10.1093/toxsci/kfr267 21984479 PMC3243748

[B10] Birch R. J. Burr N. Subramanian V. Tiernan J. P. Hull M. A. Finan P. (2022). Inflammatory bowel disease-associated colorectal cancer epidemiology and outcomes: an English population-based study. Am. J. Gastroenterol. 117 (11), 1858–1870. 10.14309/ajg.0000000000001941 36327438

[B11] Boyd R. I. Ahmad S. Singh R. Fazal Z. Prins G. S. Madak Erdogan Z. (2022). Toward a mechanistic understanding of poly- and perfluoroalkylated substances and cancer. Cancers (Basel) 14 (12), 2919. 10.3390/cancers14122919 35740585 PMC9220899

[B12] Buck R. C. Franklin J. Berger U. Conder J. M. Cousins I. T. de Voogt P. (2011). Perfluoroalkyl and polyfluoroalkyl substances in the environment: terminology, classification, and origins. Integr. Environ. Assess. Manag. 7 (4), 513–541. 10.1002/ieam.258 21793199 PMC3214619

[B13] Butenhoff J. L. Chang S. C. Olsen G. W. Thomford P. J. (2012). Chronic dietary toxicity and carcinogenicity study with potassium perfluorooctanesulfonate in Sprague Dawley rats. Toxicology 293 (1-3), 1–15. 10.1016/j.tox.2012.01.003 22266392

[B15] Centers for Disease Control and Prevention (2022). Per- and polyfluorinated substances (PFAS) factsheet. Available At: https://www.cdc.gov/biomonitoring/PFAS_FactSheet.html#:∼:text=Levels%20of%20PFAS%20in%20the%20U.S.%20Population&text=By%20measuring%20PFAS%20in%20serum,all%20of%20the%20people%20tested (Accessed May 23, 2023).

[B16] Chen Z. Yang T. Walker D. I. Thomas D. C. Qiu C. Chatzi L. (2020). Dysregulated lipid and fatty acid metabolism link perfluoroalkyl substances exposure and impaired glucose metabolism in young adults. Environ. Int. 145, 106091. 10.1016/j.envint.2020.106091 32892005 PMC8009052

[B17] Cheng H. S. Yip Y. S. Lim E. K. Y. Wahli W. Tan N. S. (2021). PPARs and tumor microenvironment: the emerging roles of the metabolic master regulators in tumor stromal-epithelial crosstalk and carcinogenesis. Cancers (Basel) 13 (9), 2153. 10.3390/cancers13092153 33946986 PMC8125182

[B18] Chi T. Wang M. Wang X. Yang K. Xie F. Liao Z. (2021). PPAR-Gamma modulators as current and potential cancer treatments. Front. Oncol. 11, 737776. 10.3389/fonc.2021.737776 34631571 PMC8495261

[B19] De Silva A. O. Armitage J. M. Bruton T. A. Dassuncao C. Heiger-Bernays W. Hu X. C. (2021). PFAS exposure pathways for humans and wildlife: a synthesis of current knowledge and key gaps in understanding. Environ. Toxicol. Chem. 40 (3), 631–657. 10.1002/etc.4935 33201517 PMC7906948

[B20] Deng P. Durham J. Liu J. Zhang X. Wang C. Li D. (2022). Metabolomic, lipidomic, transcriptomic, and metagenomic analyses in mice exposed to PFOS and fed soluble and insoluble dietary fibers. Environ. Health Perspect. 130 (11), 117003. 10.1289/EHP11360 36331819 PMC9635512

[B21] Diaz O. E. Sorini C. Morales R. A. Luo X. Frede A. Krais A. M. (2021). Perfluorooctanesulfonic acid modulates barrier function and systemic T-cell homeostasis during intestinal inflammation. Dis. Model. Mech. 14 (12), dmm049104. 10.1242/dmm.049104 34792120 PMC8713990

[B22] Ding S. Li C. Cheng N. Cui X. Xu X. Zhou G. (2015). Redox regulation in cancer stem cells. Oxid. Med. Cell. Longev. 2015, 750798. 10.1155/2015/750798 26273424 PMC4529979

[B23] Donadon M. Hudspeth K. Cimino M. Di Tommaso L. Preti M. Tentorio P. (2017). Increased infiltration of natural killer and T cells in colorectal liver metastases improves patient overall survival. J. Gastrointest. Surg. 21 (8), 1226–1236. 10.1007/s11605-017-3446-6 28536806

[B24] Dong H. Curran I. Williams A. Bondy G. Yauk C. L. Wade M. G. (2016). Hepatic miRNA profiles and thyroid hormone homeostasis in rats exposed to dietary potassium perfluorooctanesulfonate (PFOS). Environ. Toxicol. Pharmacol. 41, 201–210. 10.1016/j.etap.2015.12.009 26724606

[B25] Drew D. A. Cao Y. Chan A. T. (2016). Aspirin and colorectal cancer: the promise of precision chemoprevention. Nat. Rev. Cancer 16 (3), 173–186. 10.1038/nrc.2016.4 26868177 PMC6741347

[B26] Dunn G. P. Bruce A. T. Ikeda H. Old L. J. Schreiber R. D. (2002). Cancer immunoediting: from immunosurveillance to tumor escape. Nat. Immunol. 3 (11), 991–998. 10.1038/ni1102-991 12407406

[B27] Dzierlenga M. W. Keast D. R. Longnecker M. P. (2021). The concentration of several perfluoroalkyl acids in serum appears to be reduced by dietary fiber. Environ. Int. 146, 106292. 10.1016/j.envint.2020.106292 33395939

[B56] EFSA Panel on Contaminants in the Food Chain, Knutsen H. K. Alexander J. Barregard L. Bignami M. Bruschweiler B. (2018). Risk to human health related to the presence of perfluorooctane sulfonic acid and perfluorooctanoic acid in food. EFSA J. 16 (12), e05194. 10.2903/j.efsa.2018.5194 32625773 PMC7009575

[B28] Ekbom A. Helmick C. Zack M. Adami H. O. (1990). Ulcerative colitis and colorectal cancer. A population-based study. N. Engl. J. Med. 323 (18), 1228–1233. 10.1056/NEJM199011013231802 2215606

[B29] Elmore S. Guha N. Hsieh J. C. Y. Li K. Marder M. E. Musa M. (2021). “Proposition 65: evidence on the carcinogenicity of perfluorooctane sulfonic acid (PFOS) and its salts and transformation and degradation precursors,” in Reproductive and cancer hazard assessment branch (Sacramento, CA: Office of Environmental Health Hazard Assessment, California Environmental Protection Agency). https://oehha.ca.gov (Accessed May 23, 2023).

[B30] Fart F. Salihovic S. McGlinchey A. Gareau M. G. Oresic M. Halfvarson J. (2021). Perfluoroalkyl substances are increased in patients with late-onset ulcerative colitis and induce intestinal barrier defects *ex vivo* in murine intestinal tissue. Scand. J. Gastroenterol. 56 (11), 1286–1295. 10.1080/00365521.2021.1961306 34383611

[B31] Fenton S. E. Ducatman A. Boobis A. DeWitt J. C. Lau C. Ng C. (2021). Per- and polyfluoroalkyl substance toxicity and human health review: current state of knowledge and strategies for informing future research. Environ. Toxicol. Chem. 40 (3), 606–630. 10.1002/etc.4890 33017053 PMC7906952

[B32] Fionda C. Scarno G. Stabile H. Molfetta R. Di Censo C. Gismondi A. (2022). NK cells and other cytotoxic innate lymphocytes in colorectal cancer progression and metastasis. Int. J. Mol. Sci. 23 (14), 7859. 10.3390/ijms23147859 35887206 PMC9322916

[B33] Fragki S. Dirven H. Fletcher T. Grasl-Kraupp B. Bjerve Gutzkow K. Hoogenboom R. (2021). Systemic PFOS and PFOA exposure and disturbed lipid homeostasis in humans: what do we know and what not? Crit. Rev. Toxicol. 51 (2), 141–164. 10.1080/10408444.2021.1888073 33853480

[B34] Gao X. Schottker B. (2017). Reduction-oxidation pathways involved in cancer development: a systematic review of literature reviews. Oncotarget 8 (31), 51888–51906. 10.18632/oncotarget.17128 28881698 PMC5584299

[B35] Goodrich J. A. Walker D. Lin X. Wang H. Lim T. McConnell R. (2022). Exposure to perfluoroalkyl substances and risk of hepatocellular carcinoma in a multiethnic cohort. JHEP Rep. 4 (10), 100550. 10.1016/j.jhepr.2022.100550 36111068 PMC9468464

[B36] Greten F. R. Grivennikov S. I. (2019). Inflammation and cancer: triggers, mechanisms, and consequences. Immunity 51 (1), 27–41. 10.1016/j.immuni.2019.06.025 31315034 PMC6831096

[B37] Grivennikov S. I. (2013). Inflammation and colorectal cancer: colitis-associated neoplasia. Semin. Immunopathol. 35 (2), 229–244. 10.1007/s00281-012-0352-6 23161445 PMC3568220

[B38] Grygiel-Gorniak B. (2014). Peroxisome proliferator-activated receptors and their ligands: nutritional and clinical implications--a review. Nutr. J. 13, 17. 10.1186/1475-2891-13-17 24524207 PMC3943808

[B39] Halama N. Braun M. Kahlert C. Spille A. Quack C. Rahbari N. (2011). Natural killer cells are scarce in colorectal carcinoma tissue despite high levels of chemokines and cytokines. Clin. Cancer Res. 17 (4), 678–689. 10.1158/1078-0432.CCR-10-2173 21325295

[B40] Hibino S. Kawazoe T. Kasahara H. Itoh S. Ishimoto T. Sakata-Yanagimoto M. (2021). Inflammation-induced tumorigenesis and metastasis. Int. J. Mol. Sci. 22 (11), 5421. 10.3390/ijms22115421 34063828 PMC8196678

[B41] Hu W. Jones P. D. Celius T. Giesy J. P. (2005). Identification of genes responsive to PFOS using gene expression profiling. Environ. Toxicol. Pharmacol. 19 (1), 57–70. 10.1016/j.etap.2004.04.008 21783462

[B42] Hu W. Y. Lu R. Hu D. P. Imir O. B. Zuo Q. Moline D. (2022). Per- and polyfluoroalkyl substances target and alter human prostate stem-progenitor cells. Biochem. Pharmacol. 197, 114902. 10.1016/j.bcp.2021.114902 34968493 PMC8890783

[B43] Hussain S. P. Harris C. C. (2007). Inflammation and cancer: an ancient link with novel potentials. Int. J. Cancer 121 (11), 2373–2380. 10.1002/ijc.23173 17893866

[B44] Imai K. Matsuyama S. Miyake S. Suga K. Nakachi K. (2000). Natural cytotoxic activity of peripheral-blood lymphocytes and cancer incidence: an 11-year follow-up study of a general population. Lancet 356 (9244), 1795–1799. 10.1016/S0140-6736(00)03231-1 11117911

[B45] Imir O. B. Kaminsky A. Z. Zuo Q. Y. Liu Y. J. Singh R. Spinella M. J. (2021). Per- and polyfluoroalkyl substance exposure combined with high-fat diet supports prostate cancer progression. Nutrients 13 (11), 3902. 10.3390/nu13113902 34836157 PMC8623692

[B46] Innes K. E. Wimsatt J. H. Frisbee S. Ducatman A. M. (2014). Inverse association of colorectal cancer prevalence to serum levels of perfluorooctane sulfonate (PFOS) and perfluorooctanoate (PFOA) in a large Appalachian population. BMC Cancer 14, 45. 10.1186/1471-2407-14-45 24468211 PMC3909456

[B47] Jabeen M. Fayyaz M. Irudayaraj J. (2020). Epigenetic modifications, and alterations in cell cycle and apoptosis pathway in A549 lung carcinoma cell line upon exposure to perfluoroalkyl substances. Toxics 8 (4), 112. 10.3390/toxics8040112 33238432 PMC7711517

[B48] Jess T. Rungoe C. Peyrin-Biroulet L. (2012). Risk of colorectal cancer in patients with ulcerative colitis: a meta-analysis of population-based cohort studies. Clin. Gastroenterol. Hepatol. 10 (6), 639–645. 10.1016/j.cgh.2012.01.010 22289873

[B49] Jones P. A. Baylin S. B. (2002). The fundamental role of epigenetic events in cancer. Nat. Rev. Genet. 3 (6), 415–428. 10.1038/nrg816 12042769

[B50] Kariuki M. N. Nagato E. G. Lankadurai B. P. Simpson A. J. Simpson M. J. (2017). Analysis of sub-lethal toxicity of perfluorooctane sulfonate (PFOS) to Daphnia magna using ¹H nuclear magnetic resonance-based metabolomics. Metabolites 7 (2), 15. 10.3390/metabo7020015 28420092 PMC5487986

[B51] Kartikasari A. E. R. Huertas C. S. Mitchell A. Plebanski M. (2021). Tumor-induced inflammatory cytokines and the emerging diagnostic devices for cancer detection and prognosis. Front. Oncol. 11, 692142. 10.3389/fonc.2021.692142 34307156 PMC8294036

[B52] Keller D. S. Windsor A. Cohen R. Chand M. (2019). Colorectal cancer in inflammatory bowel disease: review of the evidence. Tech. Coloproctol. 23 (1), 3–13. 10.1007/s10151-019-1926-2 30701345

[B53] Kelly-Schuette K. A. Fomum-Mugri L. Walker J. Hoppe A. Mbanugo C. C. Nikroo N. (2022). Tumor and serum levels of per- and polyfluoroalkyl (PFAS) in hepatobiliary and gastrointestinal malignancy. Am. J. Surg. 223 (3), 514–518. 10.1016/j.amjsurg.2021.11.014 34815027

[B54] Keshavarz Shahbaz S. Koushki K. Ayati S. H. Bland A. R. Bezsonov E. E. Sahebkar A. (2021). Inflammasomes and colorectal cancer. Cells 10 (9), 2172. 10.3390/cells10092172 34571825 PMC8467678

[B55] Kim S. Thapar I. Brooks B. W. (2021). Epigenetic changes by per- and polyfluoroalkyl substances (PFAS). Environ. Pollut. 279, 116929. 10.1016/j.envpol.2021.116929 33751946

[B57] Kumari S. Badana A. K. Malla R. (2018). Reactive oxygen species: a key constituent in cancer survival. Biomark. Insights 13, 117727191875539. 10.1177/1177271918755391 PMC5808965 29449774

[B58] Kunimasa K. Goto T. (2020). Immunosurveillance and immunoediting of lung cancer: current perspectives and challenges. Int. J. Mol. Sci. 21 (2), 597. 10.3390/ijms21020597 31963413 PMC7014343

[B59] Kunovszki P. Milassin A. Gimesi-Orszagh J. Takacs P. Szanto K. Balint A. (2020). Epidemiology, mortality and prevalence of colorectal cancer in ulcerative colitis patients between 2010-2016 in Hungary - a population-based study. PLoS One 15 (5), e0233238. 10.1371/journal.pone.0233238 32407408 PMC7224530

[B60] Lai K. P. Ng A. H. Wan H. T. Wong A. Y. Leung C. C. Li R. (2018). Dietary exposure to the environmental chemical, PFOS on the diversity of gut microbiota, associated with the development of metabolic syndrome. Front. Microbiol. 9, 2552. 10.3389/fmicb.2018.02552 30405595 PMC6207688

[B61] Lakatos P. L. Lakatos L. (2008). Risk for colorectal cancer in ulcerative colitis: changes, causes and management strategies. World J. Gastroenterol. 14 (25), 3937–3947. 10.3748/wjg.14.3937 18609676 PMC2725331

[B62] Liang H. Yang M. Zeng C. Wu W. Zhao L. Wang Y. (2021). Perfluorooctane sulfonate exerts inflammatory bowel disease-like intestinal injury in rats. PeerJ 9, e10644. 10.7717/peerj.10644 33510972 PMC7798615

[B63] Liang L. Pan Y. Bin L. Liu Y. Huang W. Li R. (2022). Immunotoxicity mechanisms of perfluorinated compounds PFOA and PFOS. Chemosphere 291 (2), 132892. 10.1016/j.chemosphere.2021.132892 34780734

[B64] Lin H. W. Feng H. X. Chen L. Yuan X. J. Tan Z. (2020). Maternal exposure to environmental endocrine disruptors during pregnancy is associated with pediatric germ cell tumors. Nagoya J. Med. Sci. 82 (2), 323–333. 10.18999/nagjms.82.2.315 32581411 PMC7276419

[B65] Liu W. Zhang X. Wen Y. Anastasio M. A. Irudayaraj J. (2023). A machine learning approach to elucidating PFOS-induced alterations of repressive epigenetic marks in kidney cancer cells with single-cell imaging. Environ. Adv. 11, 100344. 10.1016/j.envadv.2023.100344

[B66] Liu Y. Li J. Ding H. Ge D. Wang J. Xu C. (2022). Perfluorooctane sulfonate (PFOS) triggers migration and invasion of esophageal squamous cell carcinoma cells via regulation of Zeb1. Drug Chem. Toxicol. 45 (6), 2804–2813. 10.1080/01480545.2021.1991775 34732098

[B67] Lu Y. Chan Y. T. Tan H. Y. Li S. Wang N. Feng Y. (2020). Epigenetic regulation in human cancer: the potential role of epi-drug in cancer therapy. Mol. Cancer 19 (1), 79. 10.1186/s12943-020-01197-3 32340605 PMC7184703

[B14] Mayer E. A. Nance K. Chen S. (2022). The gut-brain axis. Annu. Rev. Med. 73, 439–453. 10.1146/annurev-med-042320-014032 34669431

[B69] Merritt R. L. Foran C. M. (2007). Influence of persistent contaminants and steroid hormones on glioblastoma cell growth. J. Toxicol. Environ. Health A 70 (1), 19–27. 10.1080/15287390600748807 17162496

[B70] Morvan M. G. Lanier L. L. (2016). NK cells and cancer: you can teach innate cells new tricks. Nat. Rev. Cancer 16 (1), 7–19. 10.1038/nrc.2015.5 26694935

[B71] Multhoff G. Molls M. Radons J. (2011). Chronic inflammation in cancer development. Front. Immunol. 2, 98. 10.3389/fimmu.2011.00098 22566887 PMC3342348

[B73] NASEM (2022). Guidance on PFAS exposure, testing, and clinical follow-up. Washington, D.C.: The National Academies Press. 35939564

[B74] National Cancer Institute (2017). PFAS exposure and risk of cancer. Available At: https://dceg.cancer.gov/research/what-we-study/pfas (Accessed May 22, 2023).

[B75] Ngo H. T. Hetland R. B. Sabaredzovic A. Haug L. S. Steffensen I. L. (2014). *In utero* exposure to perfluorooctanoate (PFOA) or perfluorooctane sulfonate (PFOS) did not increase body weight or intestinal tumorigenesis in multiple intestinal neoplasia (Min/+) mice. Environ. Res. 132, 251–263. 10.1016/j.envres.2014.03.033 24834819

[B76] Olen O. Erichsen R. Sachs M. C. Pedersen L. Halfvarson J. Askling J. (2020). Colorectal cancer in ulcerative colitis: A scandinavian population-based cohort study. Lancet 395 (10218), 123–131. 10.1016/S0140-6736(19)32545-0 31929014

[B77] Olsen G. W. Burris J. M. Ehresman D. J. Froehlich J. W. Seacat A. M. Butenhoff J. L. (2007). Half-life of serum elimination of perfluorooctanesulfonate, perfluorohexanesulfonate, and perfluorooctanoate in retired fluorochemical production workers. Environ. Health Perspect. 115 (9), 1298–1305. 10.1289/ehp.10009 17805419 PMC1964923

[B78] Perez F. Nadal M. Navarro-Ortega A. Fabrega F. Domingo J. L. Barcelo D. (2013). Accumulation of perfluoroalkyl substances in human tissues. Environ. Int. 59, 354–362. 10.1016/j.envint.2013.06.004 23892228

[B79] Pierozan P. Cattani D. Karlsson O. (2020). Perfluorooctane sulfonate (PFOS) and perfluorooctanoic acid (PFOA) induce epigenetic alterations and promote human breast cell carcinogenesis *in vitro* . Arch. Toxicol. 94 (11), 3893–3906. 10.1007/s00204-020-02848-6 32700164 PMC7603464

[B80] Pierozan P. Karlsson O. (2018). PFOS induces proliferation, cell-cycle progression, and malignant phenotype in human breast epithelial cells. Arch. Toxicol. 92 (2), 705–716. 10.1007/s00204-017-2077-8 29063134 PMC5818598

[B81] Pierozan P. Kosnik M. Karlsson O. (2023). High-content analysis shows synergistic effects of low perfluorooctanoic acid (PFOS) and perfluorooctane sulfonic acid (PFOA) mixture concentrations on human breast epithelial cell carcinogenesis. Environ. Int. 172, 107746. 10.1016/j.envint.2023.107746 36731186

[B82] Pizzino G. Irrera N. Cucinotta M. Pallio G. Mannino F. Arcoraci V. (2017). Oxidative stress: harms and benefits for human health. Oxid. Med. Cell. Longev. 2017, 8416763. 10.1155/2017/8416763 28819546 PMC5551541

[B83] Post G. B. Cohn P. D. Cooper K. R. (2012). Perfluorooctanoic acid (PFOA), an emerging drinking water contaminant: A critical review of recent literature. Environ. Res. 116, 93–117. 10.1016/j.envres.2012.03.007 22560884

[B84] Qin Y. Gu T. Ling J. Luo J. Zhao J. Hu B. (2022). PFOS facilitates liver inflammation and steatosis: an involvement of NLRP3 inflammasome-mediated hepatocyte pyroptosis. J. Appl. Toxicol. 42 (5), 806–817. 10.1002/jat.4258 34687223

[B85] Roth K. Imran Z. Liu W. Petriello M. C. (2020). Diet as an exposure source and mediator of per- and polyfluoroalkyl substance (PFAS) toxicity. Front. Toxicol. 2, 601149. 10.3389/ftox.2020.601149 35296120 PMC8915917

[B86] Schmitt M. Greten F. R. (2021). The inflammatory pathogenesis of colorectal cancer. Nat. Rev. Immunol. 21 (10), 653–667. 10.1038/s41577-021-00534-x 33911231

[B87] Shahsavari E. Rouch D. Khudur L. S. Thomas D. Aburto-Medina A. Ball A. S. (2020). Challenges and current status of the biological treatment of PFAS-contaminated soils. Front. Bioeng. Biotechnol. 8, 602040. 10.3389/fbioe.2020.602040 33490051 PMC7817812

[B88] Sonthithai P. Suriyo T. Thiantanawat A. Watcharasit P. Ruchirawat M. Satayavivad J. (2016). Perfluorinated chemicals, PFOS and PFOA, enhance the estrogenic effects of 17β-estradiol in T47D human breast cancer cells. J. Appl. Toxicol. 36 (6), 790–801. 10.1002/jat.3210 26234195

[B89] Steenland K. Kugathasan S. Barr D. B. (2018). PFOA and ulcerative colitis. Environ. Res. 165, 317–321. 10.1016/j.envres.2018.05.007 29777922 PMC6358414

[B90] Steenland K. Winquist A. (2021). PFAS and cancer, a scoping review of the epidemiologic evidence. Environ. Res. 194, 110690. 10.1016/j.envres.2020.110690 33385391 PMC7946751

[B91] Stockholm Convention (2019). All POPs listed in the Stockholm convention. Geneva, Switzerland: United Nations Environment Programme. Available: http://chm.pops.int/TheConvention/ThePOPs/ListingofPOPs/tabid/2509/Default.aspx (Accessed May 26, 2023).

[B92] Stoffel E. M. Murphy C. C. (2020). Epidemiology and mechanisms of the increasing incidence of colon and rectal cancers in young adults. Gastroenterology 158 (2), 341–353. 10.1053/j.gastro.2019.07.055 31394082 PMC6957715

[B93] Swann J. B. Smyth M. J. (2007). Immune surveillance of tumors. J. Clin. Invest. 117 (5), 1137–1146. 10.1172/JCI31405 17476343 PMC1857231

[B94] Tan F. Jin Y. Liu W. Quan X. Chen J. Liang Z. (2012). Global liver proteome analysis using iTRAQ labeling quantitative proteomic technology to reveal biomarkers in mice exposed to perfluorooctane sulfonate (PFOS). Environ. Sci. Technol. 46 (21), 12170–12177. 10.1021/es3027715 23046066

[B95] Temkin A. M. Hocevar B. A. Andrews D. Q. Naidenko O. V. Kamendulis L. M. (2020). Application of the key characteristics of carcinogens to per and polyfluoroalkyl substances. Int. J. Environ. Res. Public Health 17 (5), 1668. 10.3390/ijerph17051668 32143379 PMC7084585

[B96] U.S. Environmental Protection Agency (2019). America’s Children and the environment. Washington, D.C.: U.S. Environmental Protection Agency. Available: https://www.epa.gov/sites/default/files/2019-07/documents/ace3_perflurochemicals_updates_11_july_2019.pdf (Accessed May, 2023).

[B97] Wang F. Lau J. K. C. Yu J. (2021a). The role of natural killer cell in gastrointestinal cancer: killer or helper. Oncogene 40 (4), 717–730. 10.1038/s41388-020-01561-z 33262461 PMC7843415

[B98] Wang F. Liu W. Jin Y. Wang F. Ma J. (2015). Prenatal and neonatal exposure to perfluorooctane sulfonic acid results in aberrant changes in miRNA expression profile and levels in developing rat livers. Environ. Toxicol. 30 (6), 712–723. 10.1002/tox.21949 24420840

[B99] Wang L. Q. Liu T. Yang S. Sun L. Zhao Z. Y. Li L. Y. (2021b). Perfluoroalkyl substance pollutants activate the innate immune system through the AIM2 inflammasome. Nat. Commun. 12 (1), 2915. 10.1038/s41467-021-23201-0 34006824 PMC8131593

[B100] Wimsatt J. H. Montgomery C. Thomas L. S. Savard C. Tallman R. Innes K. (2018). Assessment of a mouse xenograft model of primary colorectal cancer with special reference to perfluorooctane sulfonate. PeerJ 6, e5602. 10.7717/peerj.5602 30405966 PMC6216948

[B101] Wimsatt J. Villers M. Thomas L. Kamarec S. Montgomery C. Yeung L. W. (2016). Oral perfluorooctane sulfonate (PFOS) lessens tumor development in the APC(min) mouse model of spontaneous familial adenomatous polyposis. BMC Cancer 16 (1), 942. 10.1186/s12885-016-2861-5 27927180 PMC5143440

[B102] Wolf C. J. Takacs M. L. Schmid J. E. Lau C. Abbott B. D. (2008). Activation of mouse and human peroxisome proliferator-activated receptor alpha by perfluoroalkyl acids of different functional groups and chain lengths. Toxicol. Sci. 106 (1), 162–171. 10.1093/toxsci/kfn166 18713766

[B103] Xiao F. (2017). Emerging poly- and perfluoroalkyl substances in the aquatic environment: A review of current literature. Water Res. 124, 482–495. 10.1016/j.watres.2017.07.024 28800519

[B104] Yang Z. H. Dang Y. Q. Ji G. (2019). Role of epigenetics in transformation of inflammation into colorectal cancer. World J. Gastroenterol. 25 (23), 2863–2877. 10.3748/wjg.v25.i23.2863 31249445 PMC6589733

[B105] Zeng Z. Song B. Xiao R. Zeng G. Gong J. Chen M. (2019). Assessing the human health risks of perfluorooctane sulfonate by *in vivo* and *in vitro* studies. Environ. Int. 126, 598–610. 10.1016/j.envint.2019.03.002 30856447

[B106] Zhang H. Lu H. Yu L. Yuan J. Qin S. Li C. (2021). Effects of gestational exposure to perfluorooctane sulfonate on the lung development of offspring rats. Environ. Pollut. 272, 115535. 10.1016/j.envpol.2020.115535 33223333

[B107] Zhang J. Wang X. Vikash V. Ye Q. Wu D. Liu Y. (2016). ROS and ROS-mediated cellular signaling. Oxid. Med. Cell. Longev. 2016, 4350965. 10.1155/2016/4350965 26998193 PMC4779832

[B108] Zhang L. Louie A. Rigutto G. Guo H. Zhao Y. Ahn S. (2023). A systematic evidence map of chronic inflammation and immunosuppression related to per- and polyfluoroalkyl substance (PFAS) exposure. Environ. Res. 220, 115188. 10.1016/j.envres.2022.115188 36592815 PMC10044447

[B109] Zhao H. Wu L. Yan G. Chen Y. Zhou M. Wu Y. (2021). Inflammation and tumor progression: signaling pathways and targeted intervention. Signal Transduct. Target Ther. 6 (1), 263. 10.1038/s41392-021-00658-5 34248142 PMC8273155

[B110] Zhu Y. Yang D. Duan X. Zhang Y. Chen D. Gong Z. (2021). Perfluorooctane sulfonate promotes doxycycline-induced liver tumor progression in male Kras(v12) transgenic zebrafish. Environ. Res. 196, 110962. 10.1016/j.envres.2021.110962 33675800

